# Impact of lymph node dissection on clinical outcomes of intrahepatic cholangiocarcinoma: Inverse probability of treatment weighting with survival analysis

**DOI:** 10.1002/jhbp.1038

**Published:** 2021-09-16

**Authors:** Yuzo Umeda, Toshiharu Mitsuhashi, Toru Kojima, Daisuke Satoh, Kenta Sui, Yoshikatsu Endo, Masaru Inagaki, Masahiro Oishi, Takahito Yagi, Toshiyoshi Fujiwara

**Affiliations:** ^1^ Department of Gastroenterological Surgery Okayama University Graduate School of Medicine, Dentistry and Pharmaceutical Sciences Okayama Japan; ^2^ Center for Innovative Clinical Medicine Okayama University Hospital Okayama Japan; ^3^ Department of Surgery Okayama Saiseikai General Hospital Okayama Japan; ^4^ Department of Surgery Hiroshima Citizens Hospital Hiroshima Japan; ^5^ Department of Surgery Kochi Health Sciences Center Kochi Japan; ^6^ Department of Surgery Himeji Red Cross Hospital Himeji Japan; ^7^ Department of Surgery National Hospital Organization Fukuyama Medical Center Fukuyama Japan; ^8^ Department of Surgery Tottori Municipal Hospital Tottori Japan

**Keywords:** intrahepatic cholangiocarcinoma, lymph node excision, multicenter study, propensity score, retrospective studies

## Abstract

**Background:**

Lymph node metastasis (LNM) has been established as a critical risk factor for prognosis in intrahepatic cholangiocarcinoma (ICC). The clinical implications of lymph node dissection (LND) have been debated. This study aimed to clarify the prognostic impact of LND by multicenter retrospective analysis.

**Methods:**

A total of 310 ICC patients who had undergone curative resection between 2000 and 2016 were retrospectively analyzed. The prognostic impact of LND was estimated under an inverse probability of treatment weighting (IPTW) approach using propensity scores.

**Results:**

LND was performed for 224 patients (72%), with LNM pathologically confirmed in 90 patients (40%). Prognosis was poorer for patients with LNM (median survival, 16.9 months) than for those without (57.2 months; *P* < .0001). One‐, 3‐, and 5‐year overall survival rates (OS) were comparable among LND+ (81.6%, 48.0%, and 37.5%, respectively) and LND– groups (81.6%, 55.4%, and 44.6%, respectively). However, advanced tumor, as characterized by larger tumor, multinodular lesions, and serosal invasion, was significantly more frequent in the LND+ group than in the LND– group. After IPTW adjusting for imbalances, 1‐, 3‐, and 5‐year OS were better in the LND+ group (83.5%, 52.2%, and 42.8%, respectively) than in the LND– group (71.9%, 32.4%, and 23.4%, respectively; *P* = .046). LND thus showed significant prognostic impact (hazard ratio = 0.58, 95%CI = |0.39|–|0.84|, *P* = .005), especially in hilar ICC. However, peripheral ICC displayed no therapeutic benefit from LND.

**Conclusions:**

LND could have a significant role to play in improving oncologic outcomes. Therapeutic LND should be implemented on the basis of tumor location and tumor advancement.

## Graphical abstract

**Highlight**

Using the inverse probability of treatment weighting approach via propensity scores, Umeda and colleagues revealed that lymph node dissection for intrahepatic cholangiocarcinoma (ICC) showed significant prognostic impact, especially in hilar ICC, but lacked therapeutic benefit in peripheral ICC. Implementation of therapeutic lymph node dissection should be based on tumor location and advancement.

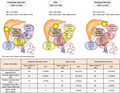

## 1 INTRODUCTION

Intrahepatic cholangiocarcinoma (ICC) is a primary liver cancer with incidence second only to hepatocellular carcinoma. ICC arises from the epithelial cells of the intrahepatic bile ducts, as either small intrahepatic ductules or large intrahepatic ducts proximal to the bifurcation of the hepatic ducts.^1^ ICC may occur in patients with normal liver or with underlying liver disease.^2^ In either clinical context, the pathology is usually classified as adenocarcinoma, although mixed hepatocellular cholangiocarcinoma also occurs, especially against a background of chronic liver disease. Reported incidences of ICC have been rising over the past two decades worldwide, including in Europe, North America, Asia, Japan, and Australia.^3^ Despite its rarity, ICC tends to be advanced or even lethal by the time of diagnosis, due to the challenges in detecting and treating the disease.

With regard to treatment for ICC, surgical resection is the only well‐established option and provides the best possibility of cure.^4^ However, only approximately 20%–40% of patients with potentially operable disease are offered surgical resection, because patients with ICC often present with large, locally advanced tumors in need of technically complex and challenging operations.^5^ Several independent factors have been associated with worsened long‐term survival, including presence of vascular invasion, symptomatic disease, regional lymph node metastasis, and multiple tumors.^6^ The incidence of lymph node metastasis (LNM) has been reported to range from 17% to 62%.^5^, ^7^, ^8^ The role of lymph node dissection (LND) at the time of surgery remains controversial, with some centers considering this procedure standard, whereas other surgeons perform LND only under select circumstances. Few studies have reported the benefits of lymphadenectomy during surgical resection for ICC.^9^ Despite the fact that node involvement is an important predictor of poor prognosis, evidence of therapeutic benefits from lymphadenectomy does not seem sufficient, and consensus is lacking about whether LND should be routinely performed.^10^

The present study aimed to identify the clinical features of LNM, including incidence of LNM, according to tumor localization, and to confirm the significance of systematic LND as a therapeutic option with curative intent.

## 2 METHODS

### 2.1 Study subjects

In this multicenter retrospective study, study subjects comprised 398 adult subjects (age range, 36‐94 years) who underwent radical resection with curative intent between January 2000 and December 2016. Clinical data for these subjects were collected from 17 medical institutions (Okayama University Hospital, Okayama Saiseikai General Hospital, Hiroshima City Hiroshima Citizens Hospital, Kochi Health Sciences Center, Himeji Red Cross Hospital, National Hospital Organization Fukuyama Medical Center, Tottori Municipal Hospital, Tenwakai Matsuda Hospital, National Hospital Organization Okayama Medical Center, Fukuyama City Hospital, Himeji St. Maria Hospital, Matsuyama Shimin Hospital, Sumitomo Besshi Hospital, Onomichi Municipal Hospital, National Hospital Organization Iwakuni Medical Center, Himeji Central Hospital, and Japanese Red Cross Kobe Hospital). Of these, 12 institutions were qualified as board‐certified training institutions for the Hepatobiliary and Pancreatic Surgery Program in Japan.^11^ Consequently, most patients were recruited from high‐volume centers which led to assured operative procedures and outcomes. Subjects meeting the following criteria were excluded: (a) non‐curative resection (residual tumor, peritoneal dissemination, or positive surgical margin [n = 13]); or (b) morphologically evident intraductal growth (n = 18); or (c) insufficient medical records for statistical analysis as described below (n = 57). After excluding those individuals who met the exclusion criteria, a total of 310 subjects were included in this study. Median follow‐up period after surgery was 25.6 months (interquartile range, 12.5‐48.9 months).

The following demographic and clinical data were reviewed through medical records to analyze predictive factors associated with LNM and significance of systematic LND: age, sex, body mass index (BMI), history of viral hepatitis, serum levels of carbohydrate antigen (CA)19‐9 and carcinoembryonic antigen (CEA), maximum tumor size, number, localization, morphology, surgical procedure, extent of LND, histological grade, vascular/serosa invasion, profiles of LNM, and postoperative complications.^12^ The definition of each pathologic factor was established based on the General Rules for the Clinical and Pathological Study of Primary Liver Cancer.^13^ With regard to localization, all ICCs were classified as hilar or peripheral based on the anatomical origin of the tumor. The anatomical location of the tumor was judged from preoperative imaging such as computed tomography or magnetic resonance imaging. The main tumors with a large proportion of tumor in contact with the hepatic hilum (between the right side of the umbilical portion of the left portal vein and the left side of the origin of the right posterior portal vein) were defined as hilar type, whereas the other tumors without these contacts were defined as peripheral type ICC.

### 2.2 Lymph node dissection

Therapeutic LND was defined as systematic lymphadenectomy including the regional lymphatic basin. Sites of lymph node were categorized according to lymphatic station around the peri‐hilum, pancreatic head, celiac axis, and lesser curvature of the stomach.^14^ With regard to LND, normal LND was defined as dissection of lymph node stations from peri‐hilum to hepatoduodenal ligament. On the other hand, extended LND was defined as normal LND plus dissection beyond the hepatoduodenal ligament, in other words, plus the common hepatic artery and posterior pancreas. Particularly with left peripheral ICCs, LND was extended to the celiac nodes and gastro‐cardiac nodes around the lesser curvature of the stomach and crus. The concept and surgical procedure for systematic LND can be browsed in the supplementary video material (Figure S1 and VIDEO S1). All harvested lymph nodes were pathologically examined to facilitate accurate disease staging after the surgeries.

### 2.3 Statistical analysis

All statistical analyses were performed using STATA/MP4 version 15.1 IC software (StataCorp LP, College Station, TX) by the Section of Medical Statistics in the Center for Innovative Clinical Medicine at Okayama University.

In the following statistical analyses, values of *P* < .050 were considered statistically significant. Continuous variables are expressed as mean or median values with interquartile range (IQR) and were compared using the Mann‐Whitney *U* test as appropriate. Categorical variables are expressed as numbers and percentages and were compared using the χ^2^ test or Fisher's exact test. Overall survival (OS) was evaluated using the Kaplan‐Meier method and compared with the log‐rank test. Multivariable logistic regression modeling was used to identify independent predictors of LNM in patients who underwent LND. Odds ratios (ORs) and 95% confidence intervals (95%CIs) were calculated.

Because of the retrospective setting, imbalances due to the intent of surgeons or institutional policy could have been present. To adjust for these imbalances in background characteristics, the inverse probability of treatment weighting (IPTW) procedure was performed, where weights were the inverse of the probabilities assigned to the actual treatment group, estimated based on the baseline demographic and clinical characteristics of patients (age, gender, body mass index, etiology [hyperlipidemia, diabetes], preoperative levels of CEA and CA19‐9, tumor factor [morphology, tumor size, uni‐ or multi‐nodular, tumor localization, vascular invasion, serosa invasion, and tumor differentiation], treatment factor [pre‐ and postoperative chemotherapy, extent of hepatectomy] using logistic regression. To avoid weighting being too heavy, weights exceeding 20 were set to 20. Even lack of only one of the aforementioned clinical variables was judged as inadequate for IPTW procedure. Thus, as described above, 57 patients were excluded from the entire primary cohort.

After confirming the hypothesis of proportional hazards, hazard ratios (HRs) and associated 95% CIs were calculated using the Cox proportional hazard model with crude analysis and IPTW. In the main analysis, the explanatory variable was set as the presence or absence of LND. In the sub‐analysis, the explanatory variable was set as no LND, extended LND, or normal LND. We also performed subgroup analysis, in which the HRs of LND were calculated according to tumor location: hilar, left peripheral, or right peripheral.

### 2.4 Ethics statement

This study was approved by the Ethics Committee of Okayama University Hospital (number 1701‐026). The need to obtain written consent was waived because of the retrospective nature of the study.

## 3 RESULTS

### 3.1 Incidence of lymph node metastasis and overall survival of the crude cohort

Clinicopathologic characteristics of the entire patient cohort are summarized in Table S1. The main morphology was mass‐forming (MF) type (76%), followed by MF and periductal‐infiltrating (PI) type (12%), and PI type (11%). Regarding surgical procedures, approximately 90% of patients underwent major hepatectomy. LND was performed for 224 patients (72%), of whom 182 patients received extended LND beyond the hepatoduodenal ligament. The indications for extended LND relied on the policy of each institution. The proportion of extended LND in patients who underwent LND was 83.4% (141/169) in the board‐certified training institutions A, 80% (28/35) in the training institutions B, and 65% (13/20) in the non‐certified training institutions, respectively (*P* = .133). In other words, high‐volume centers tended to perform extended LND. Of the 224 patients who underwent LND, LNM were pathologically confirmed in 90 patients (40%) (Table 1). The entire patient cohort was divided into an LND+ group (n = 224) and an LND– group (n = 86). Although baseline characteristics of patients with and without LND were comparable, more advanced tumors were seen in the LND+ group. That is, the LND+ group showed significantly greater tumor size (LND+ group, 4.5 cm vs LND− group, 3.3 cm; *P* = .002) and higher frequencies of multinodular lesions (LND+ group, 22.8% vs LND− group, 10.5%; *P =* .010) and serosal invasion (LND+ group, 43.3% vs LND− group, 26.7%; *P =* .020) than the LND− group. LND was performed more frequently for hilar lesions (LND+, 48.7% vs LND–, 16.3%; *P* < .001) and was accompanied by bile duct resection and vascular reconstruction in the LND+ group. As a consequence, the LND+ group required a longer operation time and showed greater blood loss than the LND– group. The postoperative morbidity rate was also higher in the LND+ group than in the LND– group (*P* = .045).

**TABLE 1:** Comparison between the LND+ group and the LND– group before and after IPTW adjustment

Variables	Before IPTW adjustment			After IPTW adjustment ^a^		
	LND+ (n = 224)	LND− (n = 86)	*P*‐value	LND+ (n = 224) Sum of weight = 310.2	LND‐ (n = 86) Sum of weight = 286.4	*P*‐value
Background factor						
Female*	98 (43.8%)	31 (36.1%)	.216	136.2 (43.9%)	123.3 (43.1%)	.928
Age*	70 (IQR 14)	72 (IQR 11)	.105	71 (IQR 14)	70 (IQR 14)	.450
Body mass index*	22 (IQR 4)	22 (IQR 4.7)	.590	22 (IQR 4.25)	23 (IQR 4)	.899
Etiology						
Hypertension	86 (38.7%)	41 (47.7%)	.154	116.5 (37.6%)	114.0 (39.8%)	.790
Hyperlipidemia*	40 (17.9%)	9 (10.5%)	.098	47.7 (15.4%)	20.5 (7.2%)	.064
Diabetes*	38 (17.0%)	27 (31.4%)	.007	66.9 (21.6%)	69.8 (24.4%)	.681
Tumor factor						
CEA (ng/mL)*^b^	3.05 (IQR 4.14)	3.55 (IQR 3.99)	.598	3.10 (IQR 3.90)	3.20 (IQR 7.44)	.914
CA19‐9 (U/mL)*^b^	53.7 (IQR 471.3)	29.4 (IQR 128.1)	.331	45.2 (IQR 280.8)	46.5 (IQR 184.6)	.174
Morphology *						
Mass‐forming (MF)	165 (73.7%)	72 (83.7%)	.145	238.2 (76.8%)	223.6 (78.1%)	.796
Periductal‐infiltrating (PI)	29 (13.0%)	6 (7.0%)		33.3 (10.7%)	21.6 (7.5%)	
MF + PI	30 (13.4%)	8 (9.3%)		38.6 (12.4%)	41.2 (14.4%)	
Tumor size (cm)*	4.5 (IQR 3.9)	3.3 (IQR 3.2)	.002	4.2 (IQR 3.5)	4.8 (IQR 4)	.778
Multi‐nodular*	51 (22.8%)	9 (10.5%)	.010	58.7 (18.9%)	56.6 (19.8%)	.920
Tumor localization*						
Hilar	109 (48.7%)	14 (16.3%)	<.001	123.6 (39.8%)	104.8 (36.6%)	.944
Peripheral left side	67 (29.9%)	40 (46.5%)		100.0 (32.2%)	95.7 (33.4%)	
Peripheral right side	48 (21.4%)	32 (37.2%)		86.6 (27.9%)	85.9 (30.0%)	
Pathology						
Vascular invasion*	128 (57.1%)	39 (45.4%)	.145	174.3 (56.2%)	146.2 (51.0%)	.842
Serosa invasion*	97 (43.3%)	23 (26.7%)	.020	134.8 (43.4%)	105.9 (37.0%)	.602
Lymph node metastasis	90 (40.2%)	‐	N/A	114.0 (36.8%)	‐	N/A
Poor grade	163 (72.8%)	64 (74.4%)	.768	217.7 (70.2%)	221.1 (77.2%)	.353
Treatment factor						
Preoperative Chemotherapy*	8 (3.6%)	0 (0.0%)	.076	8.5 (2.7%)	0 (0.0%)	N/A
Surgical procedure*						
Type of hepatectomy						
Segmentectomy	3 (1.3%)	18 (20.9%)	<.001	21.0 (6.8%)	20.7 (7.2%)	.571
Sectionectomy	18 (8.0%)	28 (32.6%)		44.8 (14.4%)	45.4 (15.9%)	
Hemihepatectomy	191 (85.3%)	39 (45.4%)		231.7 (74.7%)	217.5 (75.9%)	
Trisegmentectomy	12 (5.4%)	1 (1.2%)		12.7 (4.1%)	2.8 (1.0%)	
Bile duct resection	89 (39.7%)	6 (7.0%)	<.001	100.5 (32.4%)	41.2 (14.4%)	.037^c^
Vascular reconstruction	25 (11.2%)	2 (2.3%)	.006	27.8 (9.0%)	13.5 (4.7%)	.411
Operative time (min.)	390 (IQR 185)	280 (IQR 156)	<.001	360 (IQR 190.4)	300 (IQR 144)	.001^c^
Blood loss (mL)	820 (IQR 978)	525 (IQR 773)	.132	680 (IQR 1000)	650 (IQR 1091)	.855
Postoperative complication						
none	136 (60.7%)	61 (70.9%)	.045	200.0 (64.4%)	176.9 (61.8%)	.738
C‐D grade I‐II	54 (24.1%)	9 (10.5%)		69.3 (22.3%)	57.3 (20.0%)	
C‐D grade III‐IV	29 (13.0%)	13 (15.1%)		33.3 (10.7%)	46.6 (16.3%)	
C‐D grade V	5 (2.2%)	3 (3.5%)		7.9 (2.5%)	5.5 (1.9%)	
Postoperative chemotherapy*	107 (47.8%)	18 (20.9%)	<.001	127.8 (41.2%)	129.3 (45.1%)	.676

Note

*Variables using for calculation of propensity score.

^a^Because the weighted values were presented, the numbers of patients were not an integer.

^b^Median and interquartile range (IQR) were presented instead of mean and standard deviation.

^c^Statistical significant difference was detected after IPTW correction.

N/A: Since there was a zero cell, it was impossible to test the weighting proportion.

Abbreviations: CA19‐9, carbohydrate antigen 19‐9; C‐D, Clavien‐Dindo classification; CEA, carcinoembryonic antigen; IPTW, inverse probability of treatment weighting; LND, lymph node dissection.

**FIGURE 1:**
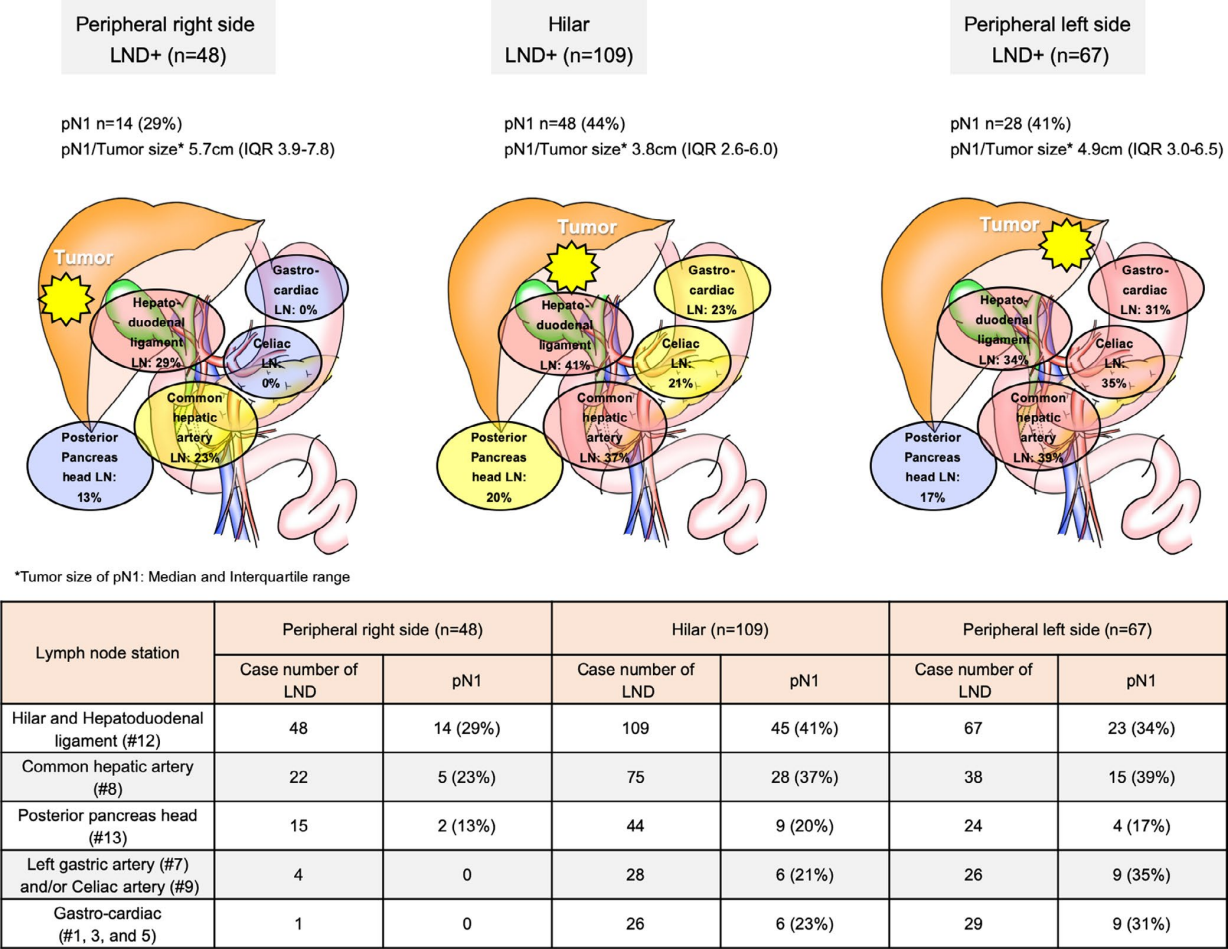
Incidence of lymph node metastasis and frequent lymph node stations according to tumor localization

In multivariate analysis of the LND+ group with identification of nodal status, morphologically evident periductal infiltration, preoperative CA19‐9 level above a cut‐off value of 118 U/mL, pathological invasion of the serosa, and moderate or poor differentiation were determined as significant risk factors for LNM (Table 2). In terms of frequent metastatic stations of LNM, some differences were identified between tumor localizations (Figure 1). In particular, hilar and left peripheral ICCs were likely to spread to gastro‐cardiac and celiac nodes beyond the hepatoduodenal ligament nodes, while right peripheral ICC showed few metastases to these nodes. Basically, lymphatic spread of right peripheral lesions tended to traverse from the hilar and hepatoduodenal ligament nodes to the nodes of the common hepatic artery and posterior pancreas head. Furthermore, median tumor size in LNM was seen in hilar ICC at 3.8 cm, followed by left peripheral ICC at 4.9 cm and right peripheral ICC at 5.7 cm.

**TABLE 2:** Logistic regression analysis to examine risk factors for lymph node metastasis

Variables		Univariable analysis				Multivariable analysis		
		Number	Odds ratio	95% CI	*P*‐value	Odds ratio	95% CI	*P*‐value
Background factor								
Sex	Male vs Female	126 vs 98	0.61	0.35‐1.03	.068	ー	ー	ー
Age	≥ 60 vs <60	199 vs 25	2.98	1.15‐9.24	.022	2.88	0.88‐11.51	.081
Body mass index	≥ 20 vs <20	165 vs 59	1.96	1.04‐3.80	.035	1.70	0.76‐3.92	.193
Hypertension	present vs absent	86 vs 138	1.21	0.69‐2.10	.503	ー	ー	ー
Hyperlipidemia	present vs absent	40 vs 184	0.85	0.40‐1.73	.661	ー	ー	ー
Diabetes	present vs absent	38 vs 186	1.11	0.53‐2.24	.771	ー	ー	ー
Tumor factor								
Morphology: Mass‐forming	vs Periductal‐infiltrating*	165 vs 59	0.42	0.22‐0.75	.004	0.29	0.12‐0.63	.002
Tumor size (cm)	≥ 4 vs 4 <	132 vs 92	1.47	0.85‐2.56	.167	ー	ー	ー
Multi‐nodular	vs Single nodular	51 vs 173	0.85	0.44‐1.61	.626	ー	ー	ー
Localization: Hilar	vs Peripheral left side predominant	109 vs 67	1.10	0.59‐2.03	.770	ー	ー	ー
	vs Peripheral right side predominant	109 vs 48	1.91	0.93‐4.05	.075	ー	ー	ー
CEA (ng/mL)	≥ 6.5 vs <6.5	52 vs 172	2.07	1.10‐3.90	.023	0.91	0.41‐1.93	.813
CA19‐9 (U/mL)	≥ 118 vs <118	85 vs 139	5.56	3.09‐10.18	<.0001	6.32	3.10‐13.52	<.0001
Pathology								
Vascular invasion	present vs absent	128 vs 96	1.05	0.60‐1.83	.865	ー	ー	ー
Serosa invasion	present vs absent	97 vs 127	1.81	1.04‐3.14	.033	2.21	1.11‐4.48	.022
Grading	mod/por vs well	163 vs 61	2.37	1.20‐4.94	.012	4.04	1.71‐10.30	.001

Note

*including mass‐forming + periductal‐infiltrating.

Abbreviations: CA19‐9, carbohydrate antigen 19‐9; CEA, carcinoembryonic antigen.

**FIGURE 2:**
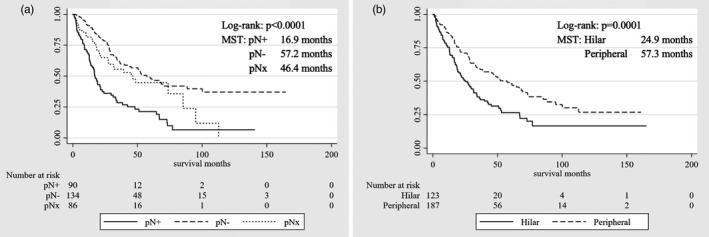
Overall survival curves after surgery in the crude cohort. (A) Status of pathological lymph node metastasis: pathological N+ versus N– vs Nx (no‐lymph node dissection). (B) Tumor localization: hilar vs peripheral

**FIGURE 3:**
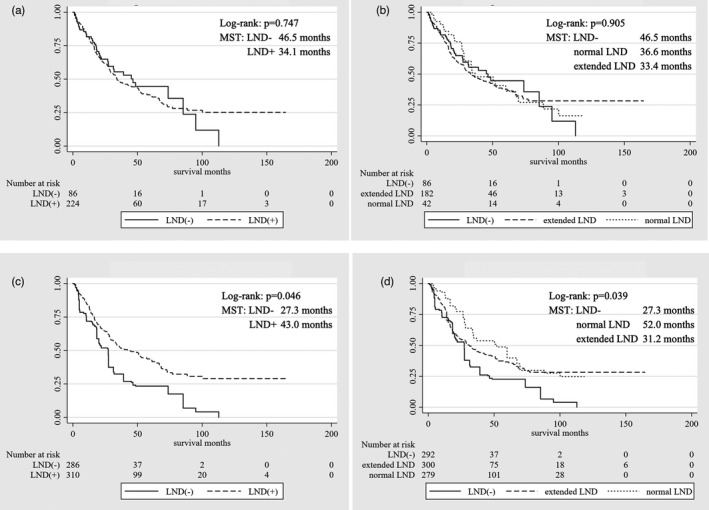
Overall survival curves after surgery in the crude cohort. (A) Status of lymph node dissection (LND): LND+ vs LND−. (B) Normal LND vs extended LND versus LND–. Overall survival curves after surgery in the IPTW adjusted cohort. (C) Status of lymph node dissection (LND): LND+ vs LND−. (D) Normal LND vs extended LND vs LND−. Figures (C) and (D) show the weighted numbers and results after adjustment by IPTW; in (C), the weights were calculated by the logistic model, and in (D), the weights were calculated by the multinomial logistic model

In survival analysis, patients with LNM showed poorer prognosis than those without LNM (median survival time [MST], 16.9 vs 57.2 months, respectively; *P* < .0001) (Figure 2A). Regarding tumor location, hilar ICC showed poorer prognosis than peripheral ICC (MST, 24.9 vs 57.3 months, respectively; *P* = .0001) (Figure 2B). Concerning the therapeutic value of LND, MST stratified by LND was 34.1 months in the LND+ group and 46.5 months in the LND– group. Similarly, 1‐, 3‐, and 5‐year OS rates were comparable among patients in the LND+ group (81.6%, 48.0%, and 37.5%, respectively) compared to the LND− group (81.6%, 55.4%, and 44.6%, respectively; *P* = .747) (Figure 3A). The prognostic impact of LND was not significant (hazard ratio [HR] = 1.06; 95% confidence interval [CI] = |0.74|–|1.51|; *P* = .747). Extended LND showed no superiority over normal LND in 1‐, 3‐, and 5‐year OS rates (normal LND, 89.8%, 51.0%, and 36.2%, vs extended LND, 79.8%, 47.3%, and 37.8%, respectively) (Table 3, Figure 3B).

**TABLE 3:** Sub‐analysis and Sub‐group analysis for prognostic impact of LND before and after IPTW adjustment

Variables		Before IPTW adjustment			After IPTW adjustment		
		Hazard ratio	95% CI	*P*‐value	Hazard ratio	95% CI	*P*‐value
Main analysis							
LND+	vs LND–	1.06	0.74‐1.51	.747	0.58	0.39‐0.84	.005
Sub‐analysis (Extent of LND)							
Extended LND+	vs LND–	1.07	0.75‐1.55	.700	0.67	0.45‐1.02	.063
Normal LND+	vs LND–	1.00	0.61‐1.66	.990	0.51	0.29‐0.90	.020
Sub‐group analysis (Tumor location)							
Hilar: LND+	vs LND–	0.65	0.35‐1.24	.192	0.45	0.25‐0.83	.011
Peripheral left side: LND+	vs LND–	0.96	0.53‐1.75	.894	0.86	0.37‐2.00	.729
Peripheral right side: LND+	vs LND–	0.97	0.49‐1.92	.938	0.52	0.25‐1.10	.089

### 3.2 Survival impact of LND among patient‐adjusted baseline characteristics by IPTW

The IPTW procedure was performed to adjust for imbalances in these retrospective settings. After IPTW adjustment, the sum of weights was 310.2 in the LND+ group and 286.4 in the LND– group. After IPTW adjusting, no variables other than bile duct resection (*P* = .037) and duration of operation (*P* = .001) remained significantly unbalanced (Table 1). Although these two variables were still significantly different after IPTW adjusting, the difference between groups was decreased. These results suggested that the balance of covariates was sufficiently improved by IPTW. As a result, background profiles and tumor‐specific characteristics of patients with and without LND were similar.

In the IPTW‐adjusted cohort, MST was longer in the LND+ group (43.0 months) than in the LND– group (27.3 months). One‐, 3‐, and 5‐year OS rates were superior in the LND+ group than in the LND‐ group (LND+, 83.5%, 52.2%, and 42.8%, vs LND−, 71.9%, 32.4%, and 23.4%, respectively; *P* = .046) (Figure 3C). LND thus showed significant prognostic impact (HR = 0.58, 95%CI = |0.39|–|0.84|, *P* = .005) (Table 3).

With regard to the extent of LND, MSTs were 52.0 months for normal LND and 31.2 months for extended LND. One‐, 3‐, and 5‐year OS rates with normal LND were comparable to those with extended LND (normal LND, 92.8%, 56.0%, and 39.8%, vs extended LND, 81.1%, 45.0%, and 36.6%, respectively; Figure 3D). A significant positive prognostic impact was seen for normal LND (vs LND−, HR = 0.51, 95%CI = |0.29|–|0.90|, *P* = .020). Although extended LND tended to show positive therapeutic value (vs LND−, HR = 0.67, 95%CI = |0.45|–|0.1.02|, *P* = .063), this was not significant. Furthermore, the significance of LND seemed to depend on tumor localization. Only hilar ICC showed significant benefit from LND (vs LND−, HR = 0.45, 95%CI = |0.25|–|0.83|, *P* = .011). On the other hand, peripheral ICC displayed no therapeutic benefit from LND (Table 3, Figure S2).

Concerning long‐surviving cases, 12 patients with pathologically confirmed LNM survived for more than 5 years after resection. Notably, all patients had undergone major hepatectomy with LND. Although nine patients showed recurrence at various sites, their survival was through treatment under a multidisciplinary approach involving resection of recurrences, chemotherapy, and radiation therapy (Table 4).

**TABLE 4:** Overview of long‐surviving cases with pathologically confirmed lymph node metastasis

#	Sex/Age	Tumor Size (cm)	Localization	Morphology	Solitary / Multiple nodule	Preoperative CA19‐9 (U/mL)	Type of hepatectomy	Bile duct resection	Vascular Reconstruction	Dissected LN*	Positive LN	Serosa/Vascular invasion	Adjuvant Chemotherapy	Recurrence	Treatment for recurrence	Outcome
1	Female/67	6.3	Peripheral left side	MF	Multiple (Unilobar)	38.4	Left hemihepatectomy	−	‐	#1, 3, 7, 8, 9, 12, 13	#7, 12	+/+	GEM + CDDP	‐	‐	5 years, alive
2	Female/73	2.0	Hilar	MF + PI	Solitary	14.0	Left hemihepatectomy	+	‐	#5, 8, 12, 13	#12	−/−	‐	‐	‐	5 years, alive
3	Male/68	7.0	Peripheral left side	MF	Solitary	4770.0	Left hemihepatectomy	−	‐	#8, 12, 13	#12	+/+	GEM	1.9 years, Lung	Chemotherapy	5.3 years, dead
4	Female/62	7.2	Peripheral left side	MF + PI	Solitary	117.8	Left hemihepatectomy	+	‐	#1, 3, 5, 7, 8, 9, 12, 13	#12	+/+	GEM	0.8 years, Lung	Chemotherapy	5.5 years, dead
5	Male/59	4.5	Hilar	PI	Solitary	462.5	Right hemihepatectomy	+	‐	#8, 12	#8, 12	±	S1	3.6 years, LN	Radiation/ Chemotherapy	5.5 years, dead
6	Female/63	10.5	Peripheral left side	MF	Solitary	40.2	Left hemihepatectomy	−	‐	#1, 3, 8, 12	#1, 12	+/+	GEM	1 year, LN	Resection	5.9 years, alive
7	Female/68	3.0	Hilar	PI	Solitary	684.0	Left hemihepatectomy	+	‐	#8, 12	#12	−/−	‐	3.6 years, LN	Chemotherapy	6 years, dead
8	Male/67	4.8	Peripheral right side	MF	Multiple (Unilobar)	16.0	Right hemihepatectomy	−	IVC	#8, 12, 13	#8	±	‐	0.8 years, Liver	Radiation/ Chemotherapy	6.1 years, dead
9	Male/75	4.3	Hilar	MF + PI	Solitary	43.7	Left hemihepatectomy	+	PV	#1, 3, 5, 7, 8, 9, 12, 13	#12	∓	‐	‐	‐	6.3 years, alive
10	Female/72	4.0	Hilar	PI	Multiple (Unilobar)	1394.0	Right hemihepatectomy	+	PV	#8, 12, 13	#8, 12	±	GEM	4.3 years, Liver	Chemotherapy	6.5 years, dead
11	Female/59	4.0	Hilar	MF + PI	Multiple (Unilobar)	2382.0	Left hemihepatectomy	+	PV, RHA	#7, 8, 12, 13	#12, Falciform ligament	+/+	‐	2.3 years, Liver	Chemotherapy/ Resection	7.3 years, dead
12	Male/67	6.5	Peripheral left side	MF	Solitary	14.8	Left hemihepatectomy	−	‐	#8, 12	#8	+/+	S1	4.0 years, Liver	Chemotherapy/ Radiation	9 years, alive

Note

*Grouping of regional lymph nodes according to the Classification of Primary Liver Cancer by the Liver Cancer Study Group of Japan. 1, Lymph nodes in the right cardinal region; 3/5, lymph nodes along the lesser curvature of the stomach; 7, lymph nodes along the left gastric artery; 8, lymph nodes along the common hepatic artery; 9, lymph nodes along the celiac artery; 12, lymph nodes in the hepatoduodenal ligament; 13, lymph nodes on the posterior surface of the pancreatic head.

Abbreviations: CDDP, cisplatin; GEM, gemcitabine; IVC, inferior vena cava; LN, lymph node; MF, mass‐forming; PI, periductal infiltrating; PV, portal vein; RHA, right hepatic artery.

## 4 DISCUSSION

ICC has been considered highly malignant, with several independent factors associated with worsened long‐term survival, including presence of vascular invasion, symptomatic disease, LNM, intrahepatic metastasis, and peritoneal dissemination. In particular, LNM is universally cited as a negative prognostic factor.^5^, ^9^, ^10^, ^15^, ^16^ ICC with LNM could be judged as an “unresectable disease” based on the systemic spread of the cancer according to the guidelines of the International Liver Cancer Association.^17^ Under such conditions of tumor biology, routine LND with curative intent has been widely performed as part of radical hepatic resection. However, few reports have referred to the positive prognostic value of LND, and survival rates have been reported as 30%–40% at 5 years postoperatively.^15^, ^18^, ^19^ In particular, LND has appeared to show no prognostic impact when the lymph node involvement is not clinically apparent. Furthermore, Li et al reported that the rate of recurrence in regional lymph nodes was only 4.9%. In other words, the prognostic value of LND has seemed limited.^20^

However, such statements have been gathering some opposition. For a start, the extent of LND has differed between reports. Further, the presence of bias in background factors and institutional policy or surgeon preferences cannot be ignored, given the retrospective settings. In this context, Kim identified a prognostic impact of LND using a propensity score‐matching method.^21^ In this report, radical surgery including adequate LND contributed to improved oncological outcomes for ICC on the basis of a propensity score‐matching method, in a study that mainly included morphological intraductal‐growth type and PI type tumors. In addition, Vitale reported that the therapeutic benefit of LND could be calculated as 5.46 months in a survival benefit simulation analysis using the SEER database.^22^ In terms of recent trends, the proportion of patients undergoing LND for ICC has been increasing year by year, particularly in Western countries.^23^ The therapeutic value of routine LND is thus a controversial but increasingly important topic.

This multi‐institutional study focused on identifying the clinical features of LNM after systemized LND and clarifying the prognostic value of LND. We also examined whether the efficacy of LND relies on tumor localization. Regarding the therapeutic value of LND, many previous studies have struggled in comparing treatment outcomes of LND, because the rarity and wide variety of clinical factors in ICC make statistical analysis difficult. Establishing a randomized controlled study would be invaluable but has not been realistic due to the relative rarity of ICC and the commonly accepted surgical strategy of LND. Initially, a propensity score‐matching method was considered for the present analysis of the impact of LND. However, this approach seemed inadequate because of severe dispersion in the distribution of actual propensity scores that lead to a serious reduction in the number of evaluable cases and a resulting loss of statistical power.^24^ In addition, in the PSM, those with very high or very low probability of receiving LND are excluded in the matching process (Figure S3). Therefore, what is estimated by PSM is not the effect of LND on the entire patient population, but only on those with a medium probability of receiving LND. IPTW, on the other hand, estimates LND by weighting. Therefore, it is possible to estimate the effect of LND on the entire patient population. Thus, there is a difference in the effect that PSM and IPTW are trying to estimate.^25^ Based on this background, the IPTW method appeared to be a more suitable analysis than a propensity score‐matching method. The clinical relevance of LND was confirmed by IPTW analysis, showing a positive prognostic impact (HR = 0.58, *P* = .005). In addition to these results, the fact that 12 survivors with LNM who survived longer than 5 years and had received radical surgery including systematic LND supported the hypothesis that LND had a positive impact. However, the utility of LND cannot be considered absolute because of some limitations to this study. Indeed, LND in the hilar region was identified as significantly beneficial in sub‐group analysis, whereas LND for peripheral ICCs exerted no significant prognostic impact on survival. Peripheral ICCs potentially have greater metastatic potential for intra‐ or extra‐hepatic spread of cancer in addition to LNM compared to hilar ICCs.

Maybe LND should only be extended up to the hepatoduodenal ligament nodes, because of the limited efficacy of extended LND and because postoperative morbidity is linked to the unfeasibility of adjuvant chemotherapy. Following the generally poor outcomes of surgery for ICCs, adjuvant therapy has recently tended to receive strong consideration for further improvement of surgical prognosis for ICC. While the clinical benefits of adjuvant therapy for ICC have 40 remained unclear, the BILCAP randomized trial recently reported adjuvant capecitabine improved overall survival for biliary tract cancer.^26^ The potential survival benefits of adjuvant chemotherapy could be associated with tumor subgroups, such as the presence of LNM and advanced tumor.^27^ From this perspective, LND is necessary for identifying nodal status.

By mapping LNM‐stratified tumor localizations, the targets of systematic LND could be clarified. Most lymph vessels of the liver flow in retrograde along the Glissonean pedicle and into lymph nodes along the hepatoduodenal ligament. The direction of LNM in extra‐hepatic sites then depends on the location of the ICC primary.^28^ In our results, hilar ICCs showed the highest ratio of LNM, at 44%, followed by left peripheral and right peripheral ICCs, as reported by previous studies. Hilar ICC reportedly shows a greater tendency to metastasize to the lymph nodes than peripheral ICC.^21^, ^29^, ^30^ In general, ICCs located in the left side of the liver spread to the gastro‐cardiac nodes around the lesser curvature of the stomach and crus. In addition to left peripheral ICCs, hilar ICCs have a higher likelihood of lymphatic spread into celiac nodes and gastro‐cardiac nodes beyond the hepatoduodenal ligament, pancreatic head, and common hepatic artery nodes. And, in our series, six patients of hilar ICC with LNM to gastro‐cardiac nodes had at least three of the four risk factors of LN metastasis, including PI components, high‐CA19‐9 level, serosa invasion, and poor differentiation. These cases were classified as hilar type based on our definition, but the average tumor size was 4.8 cm, and part of the tumor was also approaching the left peripheral. Furthermore, the CA19‐9 level was 2086 U/mL, and the vascular invasion rate was 83%, so these cases were quite advanced oncologically (data not shown). These features would result in extensive lymphatic spreading. In other words, adequate LND should be decided based on tumor location and tumor advancement.

There are some limitations to this study. This analysis focused on classical ICC and excluded narrowly defined hilar cholangiocarcinoma that was pathologically diagnosed as originating from the hilar bile ducts. However, it should be noted that there is a possibility of migration in cases where accurate differentiation is extremely difficult due to variations in imaging and diagnostic characteristics of pathologists in a retrospective, multicenter collection of cases. Regarding this issue, new molecular or other clinical evidence may resolve this in the future.

Although the significance of lymph node dissection has been debated for a long time and should be established by randomized controlled trials (RCTs), it is difficult to do so in practice and the impact can only be estimated by propensity score‐matching or simulation analysis such as IPTW, which we used in this study. Although LND has been shown to be beneficial, this result is merely statistical proof of the conventional theory. There are still many uncertainties regarding the extent and indications of LND. A well‐designed prospective study remains necessary to more fully address this issue.

## 5 CONCLUSIONS

While it has been and will continue to be difficult to conduct RCTs to prove the efficacy of LND for ICC, this is the first report to demonstrate the efficacy of LND for ICC using sufficient clinicopathological data on LNM and novel statistical method of IPTW. In addition to the essential role of LND for accurate staging to assist in decision‐making regarding adjuvant therapy, LND could have therapeutic benefits in improving patient survival. In particular, hilar ICC should be treated with extensive surgery and adequately systemized LND to achieve curative resection.

## CONFLICT OF INTEREST

The authors declare that they have no potential conflicts of interest associated with this manuscript.

## AUTHORS CONTRIBUTIONS

YU assisted with data interpretation, designed the project, and drafted the manuscript. TY and TF assisted with data interpretation. TM assisted with data interpretation and performed IPTW analysis. TK, DS, KS, EM, MI, MO, TM, TO, KH, RH, and SK provided clinicopathological data from patients.

## ETHICAL APPROVAL

This study was approved by the Ethics Committee of Okayama University Hospital (number; 1701‐026). The need to obtain written consent was waived because of the retrospective nature of the study.

## Supporting information

Fig S1

Fig S2

Fig S3

Video S1

Table S1

Legends
