# Supplementary figures and images for: Impact of lymph node dissection on clinical outcomes of intrahepatic cholangiocarcinoma: Inverse probability of treatment weighting with survival analysis
Source: J Hepatobiliary Pancreat Sci. 2021 Sep 16;29(2):217–29. doi: 10.1002/jhbp.1038 (PMC9291593; doi:10.1002/jhbp.1038)

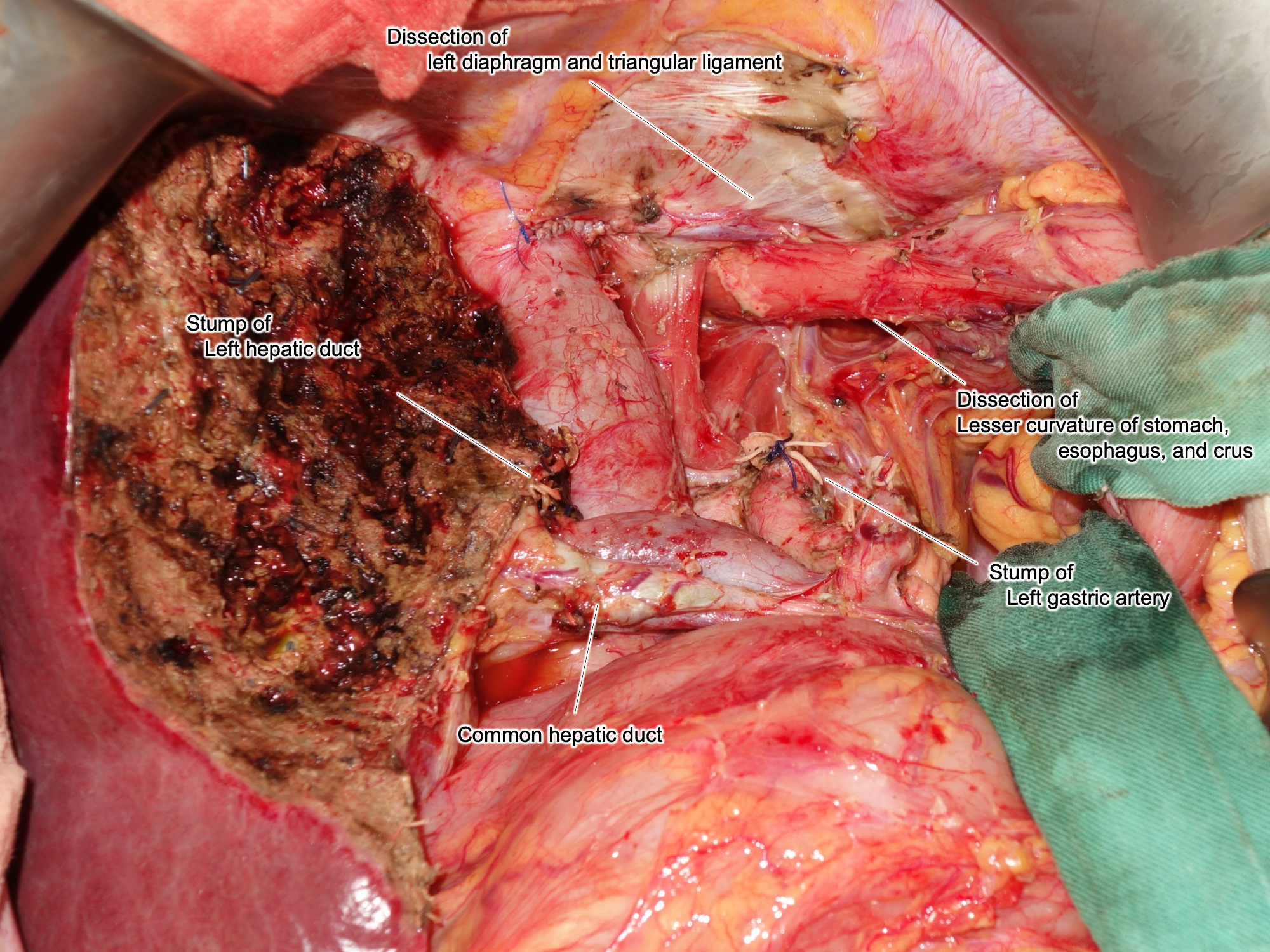

Supplement: Supplementary file 1 — Fig S1 [file JHBP-29-217-s004.tiff]

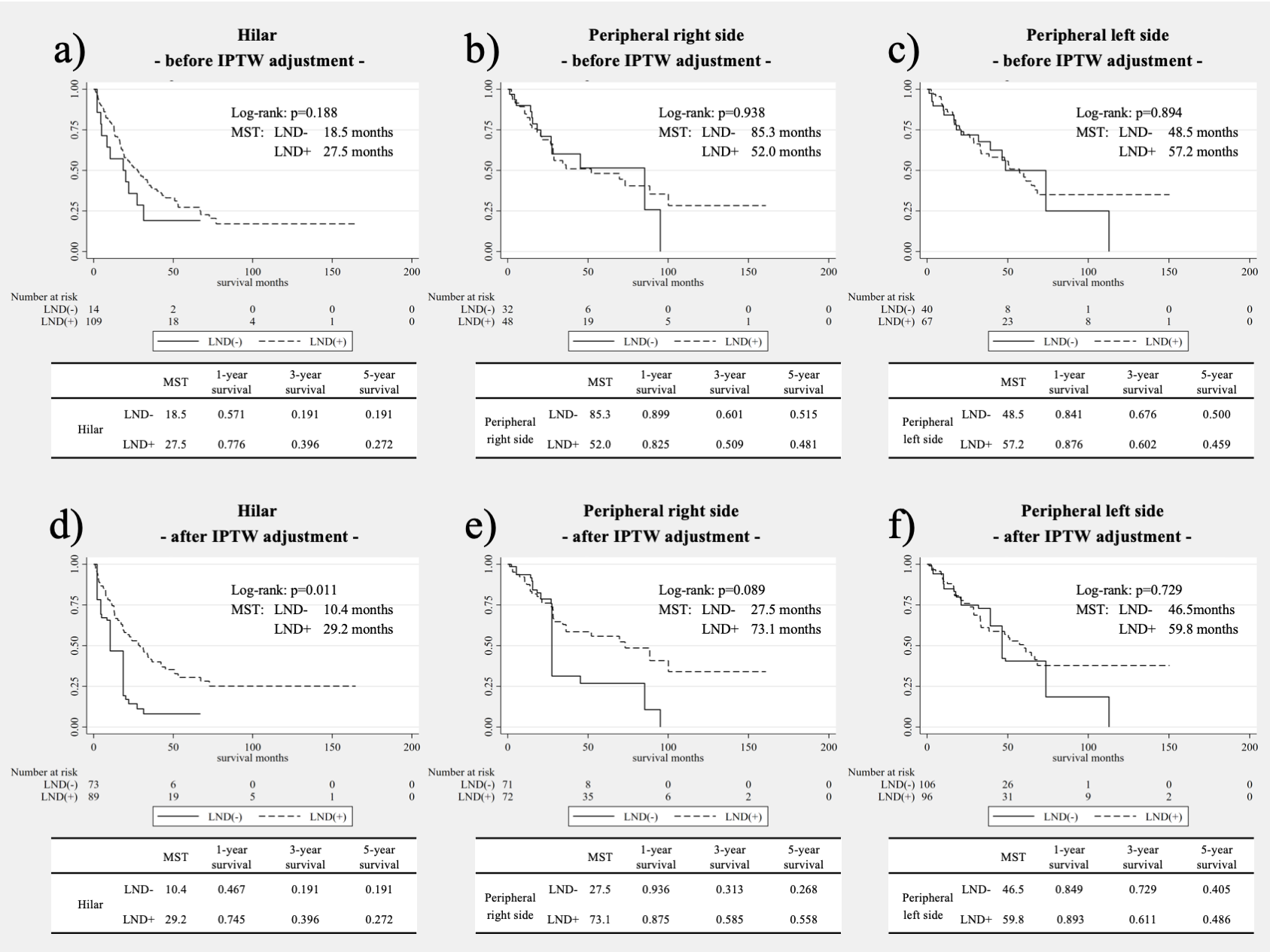

Supplement: Supplementary file 2 — Fig S2 [file JHBP-29-217-s006.tiff]

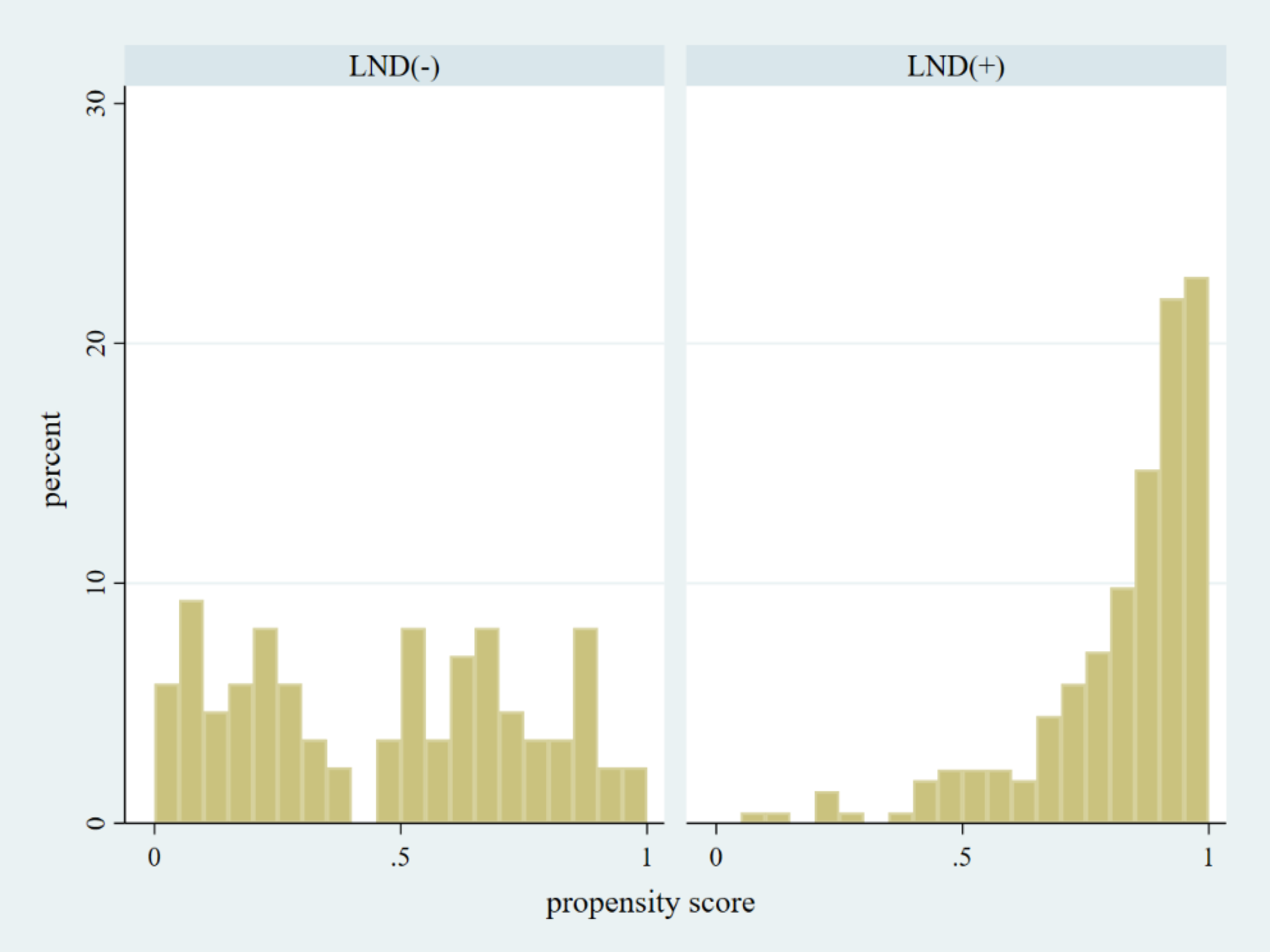

Supplement: Supplementary file 3 — Fig S3 [file JHBP-29-217-s002.tiff]
