# Supplementary material for: Impact of lymph node dissection on clinical outcomes of intrahepatic cholangiocarcinoma: Inverse probability of treatment weighting with survival analysis
Source: J Hepatobiliary Pancreat Sci. 2021 Sep 16;29(2):217–29. doi: 10.1002/jhbp.1038 (PMC9291593; doi:10.1002/jhbp.1038)
Supplement: Supplementary file 6 — Legends [file JHBP-29-217-s005.docx]

**Supplementary file**

**Table**

Clinicopathologic characteristics of the entire patient cohort.

**Figure 1 and VIDEO material.** The patient was 67 years old female diagnosed as left side predominant peripheral ICC. And she undergone left hemihepatectomy with extended LND. Pathological findings showed LNM in hepatoduodenal ligament (#12) and left gastric artery (#7) nodes. She received adjuvant chemotherapy by gemcitabine and cisplatine for 6 months. Consequently, she has been alive for 5 years without tumor recurrence.

**Figure 2.** Overall survival curves after surgery in the crude cohort. a) Hilar ICC: lymph node dissection (LND) + versus LND-. b) Peripheral right side: LND+ versus LND-. c) Peripheral left side: LND+ versus LND-. Overall survival curves after surgery in the IPTW adjusted cohort. d) Hilar ICC: LND + versus LND-. e) Peripheral right side: LND+ versus LND-. f) Peripheral left side: LND+ versus LND-.

**==== Footnote ====**

Figures d), e), and f) show the weighted numbers and results after adjustment by IPTW; the weights were calculated by the logistic model.

**Figure 3.** The distribution of propensity scores.
